# A US real-world study of treatment patterns and outcomes in localized or locally advanced prostate cancer patients

**DOI:** 10.1007/s00345-023-04680-w

**Published:** 2023-11-15

**Authors:** Stephen J. Freedland, Sandhya Nair, Xiwu Lin, Lawrence Karsh, Christopher Pieczonka, Ravi Potluri, Sabine D. Brookman-May, Suneel D. Mundle, Sarah Fleming, Neeraj Agarwal

**Affiliations:** 1 Cedars-Sinai Medical Center, Los Angeles, CA USA; 2 Durham VA Medical Center, Durham, NC USA; 3 Janssen Pharmaceutica NV, Beerse, Belgium; 4 Janssen Global Services, Horsham, PA USA; 5 The Urology Center of Colorado, Denver, CO USA; 6 Associated Medical Professionals of NY, Syracuse, NY USA; 7 Putnam Associates, HEOR & RWE, New York, NY USA; 8 Janssen Research & Development, Spring House, PA USA; 9 Department of Urology, Ludwig-Maximilians-University, Munich, Germany; 10 Janssen Global Services, Raritan, NJ USA; 11 Janssen Global Services, Titusville, NJ USA; 12 Huntsman Cancer Institute, University of Utah, Salt Lake City, UT USA

**Keywords:** Prostate cancer, Radiotherapy, Prostatectomy, Androgen deprivation therapy, Disease trajectories, Survival outcomes

## Abstract

**Purpose:**

Men with localized or locally advanced prostate cancer (LPC/LAPC) are at risk of progression after radiotherapy (RT) or radical prostatectomy (RP). Using real-world data, we evaluated patient characteristics, treatment patterns, and outcomes in LPC/LAPC.

**Methods:**

Optum claims and electronic health records (EHR) data from January 2010 to December 2021 were queried for men with LPC/LAPC who received primary RT, RP, or androgen deprivation therapy alone within 180 days after diagnosis. Survival outcomes were analyzed using descriptive statistics and Kaplan–Meier curves. Real-world overall survival (rwOS) was compared in patients with and without evidence of disease (i.e., disease recurrence, metastasis, diagnosis of castration-resistant PC) at defined time points.

**Results:**

61,772 and 62,361 men in claims and EHR cohorts met the inclusion criteria. Median follow-up was 719 and 901 days, respectively. Most men received primary RT (51.0% claims, 35.0% EHR) or RP (39.4% claims, 53.8% EHR). Survival was greatest among men treated with RP, followed by RT. Adjusted for age and comorbidity, rwOS was shorter among men with evidence of disease within 1, 3, 4, and 5 years after primary treatment than those without at the same time points.

**Conclusion:**

Real-world claims and EHR data show that survival among men with LPC/LAPC differs by primary treatment and time point of disease recurrence thereafter. Poor outcomes in men with LPC/LAPC who progress early indicate an unmet medical need for more effective primary treatment. If validated for surrogacy, no evidence of disease at specific time points could represent an intermediate efficacy endpoint in future trials.

**Supplementary Information:**

The online version contains supplementary material available at 10.1007/s00345-023-04680-w.

## Introduction

In 2020, prostate cancer (PC) was the second most common cancer and fifth most common cause of cancer death among men worldwide [1]. High-risk localized and locally advanced PC (LPC/LAPC) accounts for approximately 15% of newly diagnosed PC cases [2]. As survival rates decrease considerably following disease progression, with only about 32% of men with distant metastatic PC surviving beyond 5 years, there is an unmet need for improved treatments for LPC/LAPC [3].

Currently, a number of treatment options are recommended for LPC as a consensus on optimum management has yet to be reached, with guidance varying slightly depending on guidelines [4]. Options for low-risk LPC include watchful waiting, active surveillance, radiotherapy (RT), and radical prostatectomy (RP) [4–6]. For patients with intermediate-risk LPC and a longer life expectancy, options include RT with/without neoadjuvant/concurrent ADT or RP [4–6]. RT with neoadjuvant/adjuvant/long-term ADT with or without neoadjuvant docetaxel and RP are the recommended primary treatments for individuals with high-risk LPC/LAPC [4–6]. Observation, ADT alone, or RT are options for patients with asymptomatic disease and limited years of expected life remaining [4–6].

Real-world data (RWD) can add to the available evidence and supplement clinical trial data by addressing questions relevant to ongoing trials and therapeutic controversies, and potentially informing on future trial designs [7]. RWD and clinical trials have demonstrated that the time to metastasis-free survival (MFS) for patients with localized disease is very long, and previous work evaluating the association of early endpoints for recurrence with longer-term outcomes using clinical trial data also supports the need for earlier efficacy assessment [8–11]. We therefore aimed to determine whether we could find earlier endpoints that also correlated with poor outcomes that could, if further validated in future studies, be used as endpoints to provide a timelier treatment efficacy assessment for the LPC/LAPC patient population. Accordingly, the primary objective of this study was to leverage RWD from two US databases of insurance claims and electronic health records (EHR) to identify and describe patient characteristics, treatment patterns, and timing of disease recurrence, and their impact on survival in men with LPC/LAPC. Secondary objectives included contrasting the survival of patients with and without evidence of disease at defined time points after primary treatment. The use of two RWD sources allowed for the evaluation of the consistency of the results across datasets.

## Methods

### Overview

This retrospective, observational RWD study used information from insurance claims and EHR to identify two cohorts of men with LPC/LAPC. The analysis included clinical characteristics, treatment patterns, real-world overall survival (rwOS), real-world progression-free survival (rwPFS), real-world event-free survival (rwEFS), and real-world MFS (rwMFS). rwOS of men with and without evidence of disease at 1, 3, 4, and 5 years after receiving primary treatment was compared.

### Data sources

This report used information from two RWD sources previously used for PC research [12, 13], Optum’s Clinformatics^®^ Extended Data Mart (claims) and Optum’s PAN-Therapeutic EHR database (source descriptions in the Online resource, Supplementary Table S1), both covering the period from January 2010 to December 2021.

Although there were differences in available information in the two data sets, both included an array of demographic and clinical variables, medications, coded diagnoses, procedures, and date of death. Race and ethnicity were only available in the EHR data set. All data were deidentified, compliant with the Health Insurance Portability and Accountability Act of 1996 (HIPAA) and managed according to customer data use agreements.

### Cohort selection

Two cohorts of men with LPC/LAPC were identified, one from claims and one from EHR data. Inclusion criteria were men with an LPC/LAPC diagnosis on or after 01 January 2010 until 31 December 2021; a lookback period of ≥ 365 days available and no other primary cancers; age ≥ 18 years; and clearly defined primary treatment. As clinical data to define high-risk and locally advanced diseases were unavailable in either dataset, men were considered to have LPC/LAPC if their disease was aggressive enough to warrant treatment based on consensus of clinical opinion and experience in clinical practice by multiple experienced urologists. The type and timing of treatment were taken into account as follows. Men were considered to have LPC/LAPC if they received RT or RP within 180 days of their PC diagnosis date or had progressed to non-metastatic castration-resistant PC, metastatic castration-resistant PC (mCRPC), or metastatic castration-sensitive PC (mCSPC) within 180 days of their PC diagnosis date. Men with LPC/LAPC were also included when receiving androgen deprivation therapy (ADT) only within 180 days of their PC diagnosis date.

### Outcome definitions

Men in both cohorts were categorized into mutually exclusive groups based on primary treatment: RP, RT, or only ADT. rwOS was defined as the time from the start of primary treatment to the date of death. rwPFS was the time from primary treatment to metastasis, CRPC diagnosis, date of systemic treatment prescribed for advanced disease (CRPC or mCSPC), or death, whichever occurred first. rwEFS was the time from primary treatment to metastasis, date of systemic treatment prescribed for advanced disease (CRPC or mCSPC), death, or disease recurrence (approximated by the initiation of subsequent treatments listed in the Online resource, Supplementary Table S2), whichever occurred earlier. rwMFS was the time from the start of primary treatment to the metastasis, date of systemic treatment prescribed for advanced disease (mCRPC or mCSPC), or death. Patients who had the events for the corresponding endpoints by the data cutoff date were censored at the last enrollment end date for claims or the last activity date for EHR.

Evidence of disease was defined as disease progression (metastasis, CRPC diagnosis, date of systemic treatment prescribed for advanced disease [CRPC or mCSPC]), or receipt of any subsequent treatment. No evidence of disease was defined as no disease progression and no receipt of subsequent treatment. When estimating rwOS at 6, 7, and 8 years among men with and without evidence of disease within the specified timeframes of 1, 3, 4, and 5 years, evidence of disease was defined as disease recurrence, metastasis, CRPC diagnosis, or treatment received for CRPC/mCSPC. Patients were censored at the end of follow-up for all analyses except rwOS. For rwOS, patients were censored at the last enrollment end date (claims) or last active date (EHR).

### Statistical analysis

Summary statistics were used to describe baseline characteristics for RP, RT, and ADT groups, for both the claims and EHR cohorts. The Kruskal–Wallis test was used to compare medians of 3 groups; the log-rank test was used for survival analysis. Categorical variables are presented as counts and percentages; continuous variables are summarized with medians and interquartile ranges. Descriptive statistics were used to examine treatment sequencing and outcomes following primary treatment. Kaplan–Meier curves were used to examine the primary treatment category in relation to rwOS, rwPFS, rwEFS, and rwMFS. Among individuals who survived to 1, 3, 4, and 5 years after receiving primary treatment, hazard ratios were used to contrast rwOS among those with/without evidence of disease within each of these timeframes. For this rwOS estimate, we chose 6, 7, and 8 years because the survival among patients with LPC/LAPC is longer than that during other PC disease states and, therefore, we would expect few deaths in the first 5 years among those treated with predominantly curative intent. No evidence of disease time points of 1, 3, 4, and 5 years were used because progression at these times would have the greatest impact on OS as evidence of disease after 5 years rarely leads to death [14]. rwOS in this instance was adjusted for baseline age and level of comorbidity [Charlson Comorbidity Index (CCI)]. 95% confidence intervals are provided with estimates.

## Results

### Patient selection

61,772 and 62,361 men were identified based on the inclusion and exclusion criteria for claims and EHR cohorts, respectively (Online resource, Supplementary Fig. S1); median follow-up was 713 and 899 days in claims and EHR cohorts.

### Patient characteristics and treatment

Patient characteristics were generally similar between the claims and EHR cohorts (Online resource, Supplementary Table S3). The median age was 64 and 63 years for patients starting with RP, 71 and 70 for patients starting with RT, and 78 and 76 for patients receiving ADT alone in the claims and EHR cohorts, respectively. The proportions of patients with CCI ≥ 4 were 1.8% and 0.8% for RP, 4.4% and 2.1% for RT, and 7.1% and 2.2% for ADT alone, in the claims and EHR cohorts. The prevalence of conditions associated with poor health outcomes was generally highest among patients who received ADT alone in both the claims and EHR cohorts (Online resource, Supplementary Table S4).

In both cohorts, the majority of patients received primary treatment with either RT or RP (Online resource, Supplementary Table S5). A higher percentage of patients in the claims cohort started with RT (51.0%) compared with RP (39.4%) and ADT alone (9.6%). In the EHR cohort, a higher percentage of patients received RP (53.8%) than RT (35.0%) and ADT alone (11.3%). Secondary treatments received by patients within 180 days of primary treatment included ADT, RP (after primary RT), and RT (after primary RP).

### Real-world survival

**Fig. 1:**
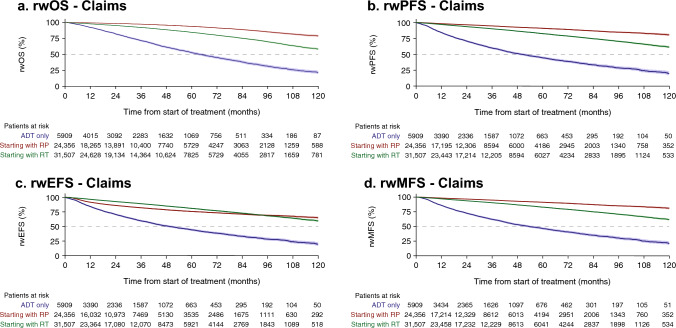
Real-world survival outcomes, rwOS (**a**), rwPFS (**b**), rwEFS (**c**), and rwMFS (**d**), among men with LPC/LAPC identified from claims data following primary treatment. *95% confidence intervals shown in shading around plotted lines. ADT* androgen deprivation therapy, *CI* confidence interval, *rwEFS* real-world event-free survival, *rwMFS* real-world metastasis-free survival, rw*OS* real-world overall survival; rw*PFS* real-world progression-free survival; *EHR* electronic health records, *LAPC* locally advanced prostate cancer, *LPC* localized prostate cancer, *RP* radical prostatectomy, *RT* radiotherapy

**Fig. 2:**
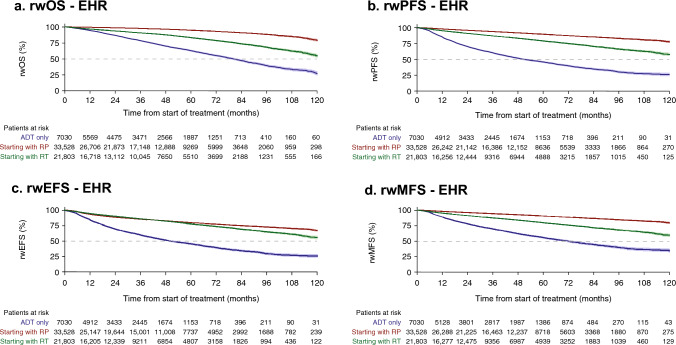
Real-world survival outcomes, rwOS (**a**), rwPFS (**b**), rwEFS (**c**), and rwMFS (**d**), among men with LPC/LAPC identified from EHR data following primary treatment. *95% confidence intervals shown in shading around plotted lines. ADT* androgen deprivation therapy, *CI* confidence interval, *rwEFS* real-world event-free survival, *rwMFS* real-world metastasis-free survival, rw*OS* real-world overall survival; rw*PFS* real-world progression-free survival; *EHR* electronic health records, *LAPC* locally advanced prostate cancer, *LPC* localized prostate cancer, *RP* radical prostatectomy, *RT* radiotherapy

In both cohorts, patients who received primary RP had the most favorable survival outcomes of rwOS, rwPFS, and rwMFS, followed by those who received primary RT and then those who received ADT alone (Figs. 1, 2, Online resource, Supplementary Table S6). Primary RP and RT demonstrated a more favorable rwEFS than ADT alone. Median OS was not reached (NR) for patients receiving primary RP; for primary RT, it was 131.4 (95% CI 129.7–NR) months and 127.8 (95% CI 121.8–NR) for claims and EHR, respectively, and for patients treated with ADT alone it was 63.6 (95% CI 61.1–66.5) and 80.1 (95% CI 77.4–82.8) months, respectively. Median rwPFS, rwEFS, and rwMFS were NR with RP and RT.

**Fig. 3:**
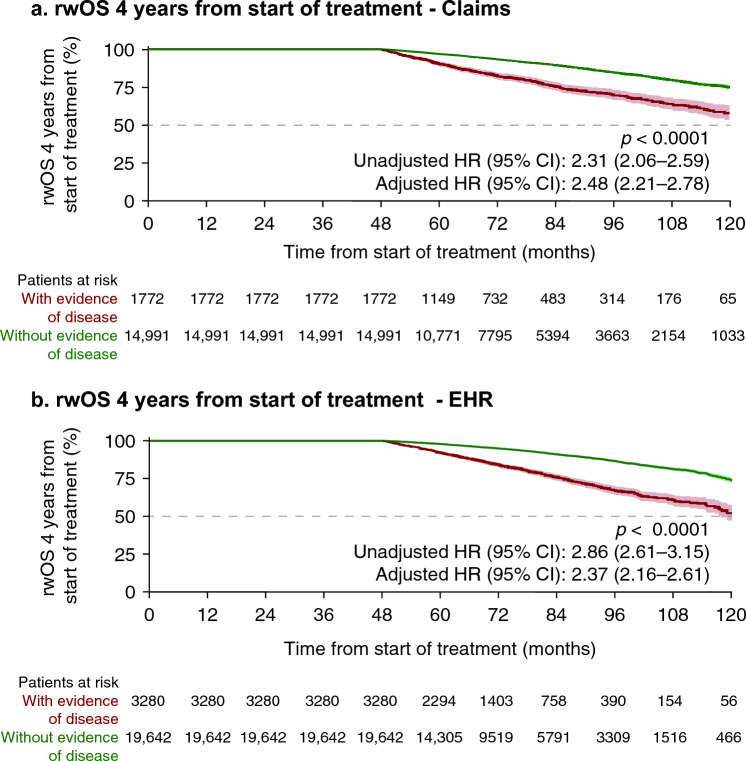
Real-world overall survival among men with LPC/LAPC identified from claims and EHR data with and without evidence of disease within 4 years of primary treatment using claims (**a**) and EHR (**b**) data. 95% confidence intervals shown in shading around plotted lines. *CI* confidence interval, *EHR* electronic health records, *HR* hazard ratio, *LAPC* locally advanced prostate cancer, *LPC* localized prostate cancer, rwOS real-world overall survival

In both cohorts, at 6, 7, and 8 years from primary treatment initiation, rwOS among men with evidence of disease within 1, 3, 4, and 5 years was less favorable than among those without; earlier evidence of disease was consistently associated with lower rwOS compared with later evidence of disease (Table 1). Adjusted mortality rates were more than two times higher among men with evidence of disease at 1, 3, 4, and 5 years than among those without (Fig. 3, Online resource, Supplementary Figs. S2 and S3). Men with evidence of disease within 4 years had adjusted mortality rates 2.48 (claims; 95% CI 2.21–2.78) and 2.37 (EHR; 95% CI 2.16–2.61) times higher than those without evidence of disease in this timeframe (Fig. 3).

**Table 1:** Real-world overall survival among men with LPC/LAPC identified from claims and EHR data who survived 1, 3, 4, and 5 years after start of primary treatment, according to evidence of disease during each time period

Time from initiation of primary treatment (years)	Survival probability (%)							
	Evidence of disease within 1 year		Evidence of disease within 3 years		Evidence of disease within 4 years		Evidence of disease within 5 years	
	Yes	No	Yes	No	Yes	No	Yes	No
Claims survival data	*n* = 1959	*n* = 43,480	*n* = 2156	*n* = 21,557	*n* = 1772	*n* = 14,991	*n* = 1332	*n* = 10,339
At 6 years	68.6	83.5	75.5	90.1	82.4	93.3	90.4	96.8
At 7 years	65.1	79.2	70.3	86.0	75.5	89.5	81.7	93.1
At 8 years	62.8	74.1	65.2	81.0	69.7	84.6	74.5	88.4
EHR survival data	*n* = 2502	*n* = 46,487	*n* = 3736	*n* = 26,802	*n* = 3280	*n* = 19,642	*n* = 2719	*n* = 13,776
At 6 years	69.5	86.2	78.0	91.9	83.9	94.9	92.1	97.5
At 7 years	62.9	81.9	70.0	87.9	75.7	91.1	82.9	94.0
At 8 years	55.2	77.4	62.3	83.4	67.2	86.7	74.7	89.7

*EHR* electronic health records, *LAPC* locally advanced prostate cancer, *LPC* localized prostate cancer

## Discussion

This real-world study of US-based patients confirms that there is an increased risk of mortality among individuals with LPC/LAPC, who experience early progression, signaling an unmet need to prevent early disease recurrence. RP followed by RT demonstrated the greatest benefit for oncological outcomes. rwOS was found to be significantly lower among men with early evidence of disease than among those without evidence of disease when analyzed at defined time points within the 5 years after primary treatment.

rwOS, rwPFS, and rwMFS were most favorable for patients with LPC/LAPC who received RP, followed by RT, and least favorable among those who received primary treatment with ADT alone. The better outcomes with RP compared with RT and ADT alone may be due to differences in patient characteristics, such as younger age and fewer comorbidities, as well as potential unknown confounders. However, current literature and treatment guidelines suggest that RP and RT are both good options for primary treatment [4–6, 15, 16]. Although patients who started on ADT alone constituted only about 10% of each cohort, their poorer outcomes were consistent across all analyses, likely impacted by older age and reduced health status, which rendered them ineligible for RT or RP.

rwOS was consistently and significantly lower in men with evidence of disease at early time points of 1, 3, 4, and 5 years than in those without, and adjusted mortality rates were consistently more than two times higher. These results suggest that early progression after primary treatment may serve as an intermediate endpoint reflecting elevated mortality risks in PC patients who are at high risk of unfavorable outcomes.

The link between early progression and survival raises questions about how time to progression may be optimized in LPC/LAPC research. There is a growing body of research focused on the development and evaluation of earlier surrogate endpoints for cancers, including PC, that could potentially mitigate some limitations inherent in current measures like MFS or OS [17–22]. Competing risks of death from causes unrelated to PC, initiation of effective subsequent treatment, and use of next-generation imaging are impacting endpoints such as OS and MFS and make it challenging to reflect clinical benefits associated with effective treatment in a reasonable timeframe. Previous work to identify intermediate endpoints has concluded that MFS is a strong surrogate for OS in men with LPC but EFS, a prostate-specific antigen-based endpoint, is not [21–23]. Ongoing clinical trials in LPC/LAPC are using the validated MFS as a primary endpoint [24, 25]. However, earlier measurable endpoints can be beneficial and may accelerate progress to identify beneficial treatments earlier, especially for disease states like LPC/LAPC, for which it takes many years to reach endpoints like MFS and OS. Other progression-related surrogate endpoints have been investigated for OS endpoints [8, 23], with EFS with prostate-specific antigen (PSA) nadir + 2 ng/mL and rising, PSA > 5 ng/mL, or PSA doubling time < 6 months ± ADT initiation and no evidence of disease identified as promising early endpoints for high-risk LPC [8]. Based on the substantially higher rwOS among men without evidence of disease described here, and if validated for surrogacy in additional LPC datasets, no evidence of disease at defined time points could be a possible intermediate endpoint for earlier efficacy assessment in future clinical trials investigating treatment for LPC/LAPC.

The worse outcomes among patients with evidence of disease within 5 years of primary treatment compared with those without evidence of disease also point to considerable unmet needs among patients with LPC/LAPC for prevention of early disease recurrence. However, in addition to earlier assessment of primary treatment efficacy, improved treatment regimens with potentially earlier secondary treatment, as well as novel treatments along with RT and RP, could improve outcomes in LPC/LAPC.

By definition, patients with high-risk LPC or LAPC are at the greatest risk for progression; yet, why some patients with LPC/LAPC progress earlier than others remains unclear. Patients in our analysis had LPC/LAPC based on receipt of primary RT or RP, or ADT alone within 180 days of diagnosis. The percentage of patients with high-risk diseases could not be clearly determined based on available data. For patients starting with RP, high-risk disease is likely if they received neoadjuvant/adjuvant ADT or RT within 180 days of RP; in our study, this equated to 10% of the claims cohort and 5% of the EHR cohort who received RP. Thus, while we excluded active surveillance patients (i.e., the lowest-risk patients), we could not confirm high-risk disease status for the rest of the patient population receiving RP or for those receiving primary RT. For those who received ADT only, high-risk disease is likely but could not be confirmed. The 5-year OS for patients with localized and regional disease (Surveillance, Epidemiology and End Results [SEER] stage) is > 99% [3]; our rwOS was lower regardless of treatment. Although we cannot exclude, due to limitations inherent in patient selection, that the present cohort could also include patients who are not high-risk per standard definition, the study cohort likely represents patients with LPC/LAPC with a higher risk of death than the general population of patients with LPC/LAPC.

Strengths of this study include the use of RWD that reflects how men with LPC/LAPC are treated in routine practice. The study also benefits from the considerable size of the data sets, including more than 120,000 men with LPC/LAPC, and the ability to establish consistent findings across insurance claims and EHR data sources. However, claims data do not describe people without insurance, and EHR data may under-represent low-income or uninsured people and they may also be subject to biases that can impact generalizability [26]. Although there are known persistent disparities in PC treatments and outcomes according to race and ethnicity with known contributing factors such as PC heritability and access to care [27, 28], they were not explored as the data were not available consistently for both cohorts.Additionally, the ADT-only group was included as it contributed a substantial number of patients from both claims and EHR data sources. However, although the ADT-only group in this study is likely heterogeneous and may contain patients who have characteristics similar to those who would follow a watchful waiting approach or have mCSPC, it was not possible to differentiate further with the available RWD. Furthermore, in the absence of RW progression data, this study utilized metastasis and treatment sequences to define and evaluate disease recurrence and evidence of disease. Finally, key variables including prostate-specific antigen and Gleason score were unavailable in both datasets and LPC/LAPC definition was based on treatment and timing.

In conclusion, oncological outcomes differed according to the type of primary treatment in men with LPC/LAPC. rwOS was significantly lower among men with early evidence of disease than those without progression at defined time points within 5 years after primary treatment. If validated in other datasets, no evidence of disease at defined time points could be considered as a possible intermediate endpoint for men with LPC/LAPC, allowing for early treatment efficacy assessment. These results also complement ongoing clinical trials, adding to a growing body of literature demonstrating the persistent unmet need for new more effective treatments for men with LPC/LAPC.

### Supplementary Information

Below is the link to the electronic supplementary material.

Supplementary file 1 (DOCX 323 KB)

## Data Availability

The data for this study were obtained from Optum’s Clinformatics® Extended Data Mart database and Optum’s PAN-Therapeutic electronic health records database under license from Optum. Information on accessing these databases can be found online (https://www.optum.com/business/life-sciences/real-world-data.html).
