# Supplementary material for: A US real-world study of treatment patterns and outcomes in localized or locally advanced prostate cancer patients
Source: World J Urol. 2023 Nov 15;41(12):3535–42. doi: 10.1007/s00345-023-04680-w (PMC10693516; doi:10.1007/s00345-023-04680-w)
Supplement: Supplementary file 1 — (DOCX 323 KB) [file 345_2023_4680_MOESM1_ESM.docx]

**Online Resource:**

**A US real-world study of treatment patterns and outcomes in localized or locally advanced prostate cancer patients**

**Stephen J. Freedland • Sandhya Nair • Xiwu Lin • Lawrence Karsh • Christopher Pieczonka • Ravi Potluri • Sabine D. Brookman-May • Suneel D. Mundle • Sarah Fleming • Neeraj Agarwal**

**Supplementary Fig. 1** Patient identification


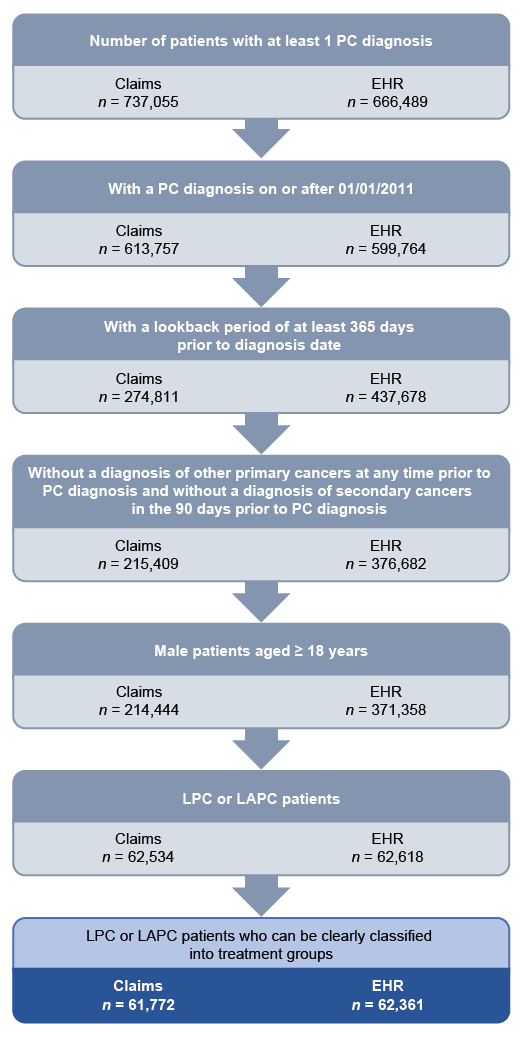


*EHR* electronic health records, *LAPC* locally advanced prostate cancer, *LPC* localized prostate cancer, *PC* prostate cancer

**Supplementary Table 1** Comparison of Optum’s claims and EHR databases^1,2^

|  | | Optum’s Clinformatics® Extended Data Mart database (claims) | Optum’s PAN-Therapeutic EHR database (EHR) |
| --- | --- | --- | --- |
| General description | | - Derived from a database of administrative health claims for members of large commercial and Medicare Advantage health plans. - Adjudicated US administrative health claims database describing people with private health insurance | - Derived from dozens of healthcare provider organizations in the United States |
| Country | | - United States | - United States |
| Patient population | | - Representative of commercial claims patients (aged 0–65 years) - Some Medicare patients (aged 65+ years) - Patients in the database are capped at age 90 | - > 80 million patients - At least 7 million patients in each US Census region - > 7700 hospitals and clinics |
|  |  | - Overlap of patients between the databases (estimated to be 10–26% of EHR) | |
| Data source | | - Closed (sourced from payers) - Complete record of healthcare encounters of a patient) insurance claims database | - Data sourced from multiple EHR vendors |
| Payer | | - Single payer (United Healthcare) | - NA |
| Available data | General | - Demographic variables, clinical variables, coded diagnoses and procedures - Information from administrative claims processed from inpatient, emergency department, outpatient medical services and dispensed prescriptions - Information from outpatient lab result tests processed by large national laboratory vendors that participate in data exchange with Optum | - Demographic variables, race/ethnicity, clinical variables, coded diagnoses and procedures - Information on outpatient visits, diagnostic procedures, medications, laboratory results, hospitalizations, clinical notes and patient outcomes - No curated data from patient charts - Contains structured data created from physician notes using Optum’s proprietary natural language processing algorithms |
|  | Drug | - Available from actual prescription fills at pharmacies and procedures undertaken | - Available by way of prescriptions written (but not of actual fills) and procedures undertaken |
|  | Cost | - Standardized costs corresponding to allowed amounts but not actual costs | - No cost data |
|  | Death | - Date of death (month and year only) for members with both medical and pharmacy coverage from the Social Security Death Master File | - Date of death |

*EHR* electronic health record

^1^ Khera R, Schuemie MJ, Lu Y, et al (2022) Large-scale evidence generation and evaluation across a network of databases for type 2 diabetes mellitus (LEGEND-T2DM): a protocol for a series of multinational, real-world comparative cardiovascular effectiveness and safety studies. BMJ Open 12:e057977. <https://doi.org/10.1136/bmjopen-2021-057977>.

^2^ Choi YC, Zhang D, Tyczynski JE (2021) comparison between health insurance claims and electronic health records (ehrs) for metastatic non-small-cell lung cancer (nsclc) patient characteristics and treatment patterns: a retrospective cohort study. Drugs Real World Outcomes 8:577-587. <https://doi.org/10.1007/s40801-021-00269-0>.

**Supplementary Table 2** Definition of disease recurrence dates

RP 🡪 ADT (> 180 days) - Start of ADT as progression date​

RP 🡪 RT (> 180 days) - Start of RT as progression date​

RP 🡪 ADT + RT (> 90 days) - Start of earlier of ADT and RT as progression date​

RT 🡪 ADT (> 180 days) - Start of ADT as progression date​

RT 🡪 RP (> 180 days) - Start of RP as progression date

*ADT* androgen deprivation therapy, *RP* radical prostatectomy, *RT* radiotherapy

**Supplementary Table 3** Age, days to LPC/LAPC progression, comorbidity and demographic characteristics of two cohorts of men with LPC/LAPC based on claims and EHR data

|  | Claims | | | | EHR | | | |
| --- | --- | --- | --- | --- | --- | --- | --- | --- |
|  | Overall  (*N* *=*61,772) | Starting with RP (*n* *=*24,356) | Starting with RT (*n* *=*31,507) | ADT only  (*n =*5909) | Overall  (*n =*62,361) | Starting with RP (*n =*33,528) | Starting with RT (*n =*21,803) | ADT only  (*n =*7030) |
| Median age at diagnosis, years (IQR) | 69 (63–74) | 64 (59–69) | 71 (67–76) | 78 (71–83) | 66 (61–72) | 63 (58–68) | 70 (64–75) | 76 (69–80) |
| *p* Value | – | *p* < 0.0001 | | | – | *p* < 0.0001 | | |
| Median time from diagnosis to progression^a^, days (IQR) | 713 (290–1411) | 631 (259–1298) | 825 (353–1545) | 496 (156–1166) | 899 (351–1698) | 950 (365–1746) | 890 (353–1705) | 700 (293–1408) |
| *p* Value | – | *p* < 0.0001 | | | – | *p* < 0.0001 | | |
| CCI—n (%) |  |  |  |  |  |  |  |  |
| 0 | 40,021 (64.8) | 17,764 (72.9) | 19,249 (61.1) | 3008 (50.9) | 49,899 (80.0) | 27,692 (82.6) | 16,805 (77.1) | 5402 (76.8) |
| 1 | 9442 (15.3) | 3070 (12.6) | 5240 (16.6) | 1132 (19.2) | 5955 (9.5) | 3087 (9.2) | 2155 (9.9) | 713 (10.1) |
| 2 | 7469 (12.1) | 2462 (10.1) | 4115 (13.1) | 892 (15.1) | 4349 (7.0) | 2033 (6.1) | 1761 (8.1) | 555 (7.9) |
| 3 | 2597 (4.2) | 615 (2.5) | 1525 (4.8) | 457 (7.7) | 1272 (2.0) | 448 (1.3) | 619 (2.8) | 205 (2.9) |
| 4+ | 2243 (3.6) | 445 (1.8) | 1378 (4.4) | 420 (7.1) | 886 (1.4) | 268 (0.8) | 463 (2.1) | 155 (2.2) |
| *p* Value | – | *p* < 0.0001 | | | – | *p* < 0.0001 | | |
| Race—n (%) |  |  |  |  |  |  |  |  |
| Caucasian | – | – | – | – | 51,023 (81.8) | 27,880 (83.2) | 17,457 (80.1) | 5686 (80.9) |
| African American | – | – | – | – | 8174 (13.1) | 4091 (12.2) | 3163 (14.5) | 920 (13.1) |
| Asian | – | – | – | – | 533 (0.9) | 281 (0.8) | 175 (0.8) | 77 (1.1) |
| Other/Unknown | – | – | – | – | 2631 (4.2) | 1276 (3.8) | 1008 (4.6) | 347 (4.9) |
| *p* Value | – | | | | – | *p* < 0.0001 | | |
| Ethnicity—n (%) |  |  |  |  |  |  |  |  |
| Non-Hispanic | – | – | – | – | 56,209 (90.1) | 30,503 (91.0) | 19,444 (89.2) | 6262 (89.1) |
| Hispanic | – | – | – | – | 1585 (2.5) | 925 (2.8) | 497 (2.3) | 163 (2.3) |
| Unknown | – | – | – | – | 4567 (7.3) | 2100 (6.3) | 1862 (8.5) | 605 (8.6) |
| *p* Value | – | | | | – | *p* < 0.0001 | | |
| US region—n (%) |  |  |  |  |  |  |  |  |
| Northeast | 7138 (11.6) | 2377 (9.8) | 4121 (13.1) | 640 (10.8) | 8477 (13.6) | 4230 (12.6) | 3231 (14.8) | 1016 (14.5) |
| West | 13,218 (21.4) | 4888 (20.1) | 6755 (21.4) | 1575 (26.7) | 5861 (9.4) | 3101 (9.2) | 1807 (8.3) | 953 (13.6) |
| Midwest | 14,652 (23.7) | 6888 (28.3) | 6473 (20.5) | 1291 (21.8) | 34,078 (54.6) | 18,651 (55.6) | 12,092 (55.5) | 3335 (47.4) |
| South | 26,619 (43.1) | 10,104 (41.5) | 14,122 (44.8) | 2393 (40.5) | 12,261 (19.7) | 6605 (19.7) | 4175 (19.1) | 1481 (21.1) |
| Unknown | 145 (0.2) | 99 (0.4) | 36 (0.1) | 10 (0.2) | 1684 (2.7) | 941 (2.8) | 498 (2.3) | 245 (3.5) |
| *p* Value | – | *p*< 0.0001 | | | – | *p*< 0.0001 | | |

^a^Time from LAPC diagnosis to progression (including disease recurrence) or end of follow-up

There is no information regarding race and ethnicity in Optum claims

*ADT* androgen deprivation therapy, *CCI* Charlson Comorbidity Index, *EHR* electronic health records, *IQR* interquartile range, *LAPC* locally advanced prostate cancer, *LPC* localized prostate cancer, *RP* radical prostatectomy, *RT* radiotherapy

**Supplementary Table** **4** Comorbidities of two cohorts of men with LPC/LAPC based on claims and EHR data

| Comorbidity—n.(%) | Claims | | | | | EHR | | | |
| --- | --- | --- | --- | --- | --- | --- | --- | --- | --- |
|  | Overall  (*N =*61,772) | Starting with RP  (*n =*24,356) | Starting with RT  (*n =*31,507) | ADT only  (*n =*5909) | Overall  (*N =*62,361) | | Starting with RP  (*n =*33,528) | Starting with RT  (*n =*21,803) | ADT only  (*n =*7030) |
| Hypertension | 39,400 (64.1) | 14,006 (57.6) | 21,265 (67.9) | 4129 (70.9) | 24,879 (46.0) | | 13,690 (44.4) | 8661 (49.5) | 2528 (43.9) |
| Chronic pulmonary disease | 7630 (12.4) | 2362 (9.7) | 4289 (13.7) | 979 (16.8) | 5007 (9.3) | | 2423 (7.9) | 1982 (11.3) | 602 (10.4) |
| Cardiac arrhythmia | 9991 (16.3) | 3568 (14.7) | 5108 (16.3) | 1315 (22.6) | 7452 (13.8) | | 4243 (13.7) | 2373 (13.6) | 836 (14.5) |
| Diabetes | 15,595 (25.4) | 4628 (19.0) | 9053 (28.9) | 1914 (32.9) | 8475 (15.7) | | 4033 (13.1) | 3355 (19.2) | 1087 (18.9) |
| Fluid and electrolyte disorders | 3212 (5.2) | 856 (3.5) | 1789 (5.7) | 567 (9.7) | 1890 (3.5) | | 900 (2.9) | 732 (4.2) | 258 (4.5) |
| Peripheral vascular disorders | 6994 (11.4) | 1723 (7.1) | 4179 (13.3) | 1092 (18.7) | 2910 (5.4) | | 1188 (3.8) | 1299 (7.4) | 423 (7.3) |
| Depression | 3964 (6.4) | 1560 (6.4) | 1979 (6.3) | 425 (7.3) | 2893 (5.3) | | 1693 (5.5) | 931 (5.3) | 269 (4.7) |
| Hypothyroidism | 4988 (8.1) | 1650 (6.8) | 2719 (8.7) | 619 (10.6) | 2465 (4.6) | | 1337 (4.3) | 809 (4.6) | 319 (5.5) |
| Congestive heart failure | 3621 (5.9) | 708 (2.9) | 2205 (7.0) | 708 (12.2) | 1768 (3.3) | | 566 (1.8) | 853 (4.9) | 349 (6.1) |
| Weight loss | 1240 (2.0) | 281 (1.2) | 695 (2.2) | 264 (4.5) | 635 (1.2) | | 238 (0.8) | 282 (1.6) | 115 (2.0) |
| Renal failure | 6320 (10.3) | 1421 (5.8) | 3734 (11.9) | 1165 (20.0) | 2841 (5.2) | | 1060 (3.4) | 1244 (7.1) | 537 (9.3) |
| Valvular disease | 4149 (6.7) | 1290 (5.3) | 2283 (7.3) | 576 (9.9) | 2108 (3.9) | | 986 (3.2) | 824 (4.7) | 298 (5.2) |
| Obesity | 6722 (10.9) | 2614 (10.8) | 3572 (11.4) | 536 (9.2) | 5927 (11.0) | | 3710 (12.0) | 1835 (10.5) | 382 (6.6) |
| Other neurological disorders | 1830 (3.0) | 450 (1.9) | 1036 (3.3) | 344 (5.9) | 1086 (2.0) | | 442 (1.4) | 455 (2.6) | 189 (3.3) |
| Liver disease | 4684 (7.6) | 1826 (7.5) | 2459 (7.8) | 399 (6.8) | 2446 (4.5) | | 1357 (4.4) | 900 (5.1) | 189 (3.3) |
| Pulmonary circulation disorders | 988 (1.6) | 232 (1.0) | 614 (2.0) | 142 (2.4) | 577 (1.1) | | 212 (0.7) | 284 (1.6) | 81 (1.4) |
| Rheumatoid arthritis/collagen | 1563 (2.5) | 516 (2.1) | 869 (2.8) | 178 (3.1) | 810 (1.5) | | 436 (1.4) | 294 (1.7) | 80 (1.4) |
| Deficiency anemia | 1944 (3.2) | 457 (1.9) | 1,158 (3.7) | 329 (5.6) | 650 (1.2) | | 236 (0.8) | 289 (1.7) | 125 (2.2) |
| Alcohol abuse | 1082 (1.8) | 390 (1.6) | 581 (1.9) | 111 (1.9) | 985 (1.8) | | 551 (1.8) | 338 (1.9) | 96 (1.7) |
| Coagulopathy | 1738 (2.8) | 501 (2.1) | 997 (3.2) | 240 (4.1) | 1003 (1.9) | | 465 (1.5) | 388 (2.2) | 150 (2.6) |
| Drug abuse | 640 (1.0) | 203 (0.8) | 368 (1.2) | 69 (1.2) | 431 (0.8) | | 252 (0.8) | 133 (0.8) | 46 (0.8) |
| Psychoses | 279 (0.5) | 74 (0.3) | 147 (0.5) | 58 (1.0) | 165 (0.3) | | 61 (0.2) | 70 (0.4) | 34 (0.6) |
| Paralysis | 261 (0.4) | 50 (0.2) | 166 (0.5) | 45 (0.8) | 127 (0.2) | | 37 (0.1) | 70 (0.4) | 20 (0.3) |
| Peptic ulcer disease excluding bleeding | 293 (0.5) | 83 (0.3) | 167 (0.5) | 43 (0.7) | 227 (0.4) | | 90 (0.3) | 114 (0.7) | 23 (0.4) |
| Blood loss anemia | 331 (0.5) | 79 (0.3) | 191 (0.6) | 61 (1.0) | 153 (0.3) | | 61 (0.2) | 64 (0.4) | 28 (0.5) |
| AIDS/HIV | 182 (0.3) | 76 (0.3) | 85 (0.3) | 21 (0.4) | 102 (0.2) | | 57 (0.2) | 39 (0.2) | 6 (0.1) |
| Solid tumor | 1351 (2.2) | 385 (1.6) | 818 (2.6) | 148 (2.5) | 1155 (2.1) | | 394 (1.3) | 650 (3.7) | 111 (1.9) |
| Lymphoma | 123 (0.2) | 31 (0.1) | 85 (0.3) | 7 (0.1) | 131 (0.2) | | 32 (0.1) | 84 (0.5) | 15 (0.3) |

Listed comorbidities are from the Elixhauser comorbidity table; comorbidities have been evaluated in the 180 days prior to start of respective settings

*ADT* androgen deprivation therapy, *EHR* electronic health records, *LAPC* locally advanced prostate cancer, *LPC* localized prostate cancer, *RP* radical prostatectomy, *RT* radiotherapy

**Supplementary Table 5** Treatment trajectories in two cohorts of men with LPC/LAPC based on claims and EHR data

| Treatment sequence | Patients—no. (%) | | Time from start of first treatment to start of second treatment, days |
| --- | --- | --- | --- |
|  | Claims  (*N =*61,772) | EHR  (*N =*62,361) |  |
| Starting with RT^a^ | 31,507 (51.0) | 21,803 (35.0) | – |
| Starting with RP | 24,356 (39.4) | 33,528 (53.8) | – |
| RP only | 18,885 (30.6) | 28,802 (46.2) | – |
| ADT (neoadjuvant) 🡪 RP | 531 (0.9) | 270 (0.4) | 0–180 and > 180 |
| RT^b^ 🡪 RP | 217 (0.4) | 78 (0.1) | 0-180 |
| RP 🡪 ADT | 180 (0.3) | 387 (0.6) | > 180 |
| RP 🡪 RT | 1177 (1.9) | 1522 (2.4) | > 180 |
| RP 🡪 ADT+RT | 1461 (2.4) | 911 (1.5) | > 90 |
| RP 🡪 RT | 852 (1.4) | 639 (1.0) | 0–180 |
| RP 🡪 ADT | 363 (0.6) | 551 (0.9) | 0–180 |
| RP 🡪 ADT+RT | 690 (1.1) | 368 (0.6) | 0–90 |
| ADT only | 5909 (9.6) | 7030 (11.3) | – |

^a^Treatment trajectories (time from start of first treatment to start of second treatment) were as follows: RT only, ADT+RT 🡪 RP (0-180 days), ADT 🡪 RT (>180 days), RT 🡪 salvage RP (> 180 days), RT 🡪 ADT (0–180 days or salvage ADT occurring after >180 days), and ADT+RT (RT received within 0-180 days of ADT was considered as concurrent treatment)

^b^Patients changed their mind regarding primary treatment and changed to RP shortly after RT initiation

*ADT* androgen deprivation therapy, *EHR* electronic health records, *LAPC* locally advanced prostate cancer, *LPC* localized prostate cancer, *RP* radical prostatectomy, *RT* radiotherapy

**Supplementary Table 6** Real-World oncological outcomes following primary treatment for LPC/LAPC

| Oncological outcome | Primary treatment  Median (95% CI), months | | |
| --- | --- | --- | --- |
|  | Starting with RP | Starting with RT | ADT alone |
| Claims | *n* = 24,356 | *n =*31,507 | *n =*5909 |
| Median rwOS | NR | 131.4 (129.7–NR) | 63.6 (61.1–66.5) |
| Median rwPFS | NR | NR | 50.1 (46.9–53.3) |
| Median rwEFS | NR | NR | 50.1 (46.9–53.3) |
| Median rwMFS | NR | NR | 53.3 (50.2–57.0) |
| EHR | *n =*33,528 | *n =*21,803 | *n =*7030 |
| Median rwOS | NR | 127.8 (121.8–NR) | 80.1 (77.4–82.8) |
| Median rwPFS | NR | NR | 50.9 (48.6–54.0) |
| Median rwEFS | NR | NR | 50.9 (48.6–54.0) |
| Median rwMFS | NR | NR | 71.4 (67.7–74.5) |

*ADT* androgen deprivation therapy, *EHR* electronic health records, *CI* confidence interval, *LAPC* locally advanced prostate cancer, *LPC* localized prostate cancer, *NR* not reported, rw*OS* real-world overall survival, *RP* radical prostatectomy, *RT* radiotherapy, *rwEFS* real-world event-free survival, rw*MFS* real-world metastasis-free survival, *rwPFS* real-world progression-free survival

**Supplementary Fig. 2** Real-world overall survival among men with LPC/LAPC identified from claims data with and without evidence of disease within (a) 1, (b) 3 and (c) 5 years of primary treatment, with 95% confidence intervals


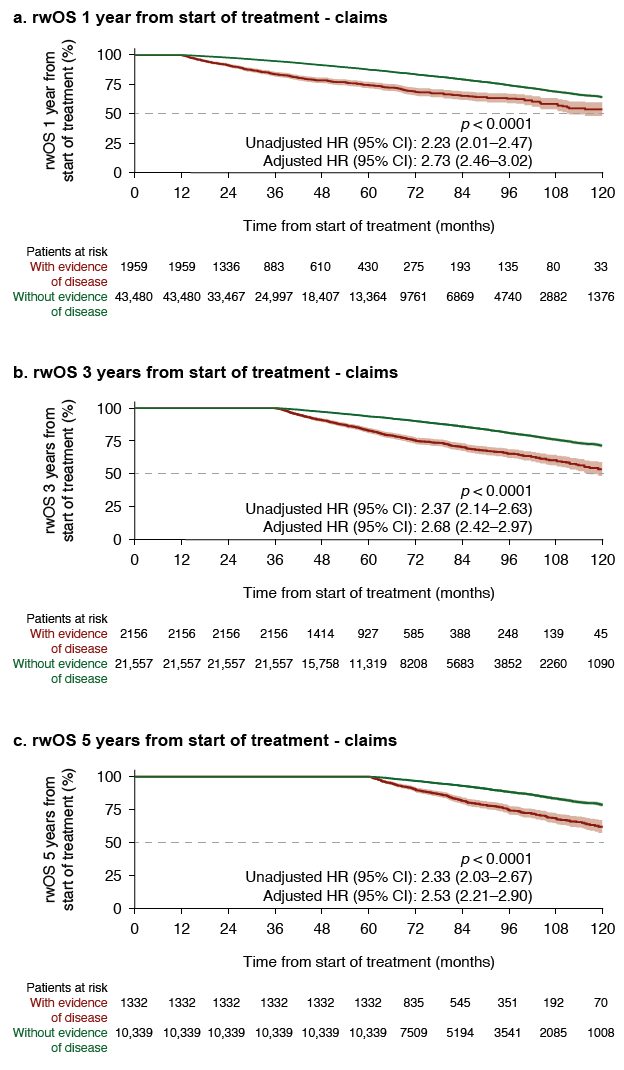


95% confidence intervals shown in shading around plotted lines

*CI* confidence interval, *HR* hazard ratio, *LAPC* locally advanced prostate cancer, *LPC* localized prostate cancer, rw*OS* real-world overall survival

**Supplementary Fig. 3** Real-world overall survival among men with LPC/LAPC identified from EHR data with and without evidence of disease within (a) 1, (b) 3 and (c) 5 years of primary treatment, with 95% confidence intervals


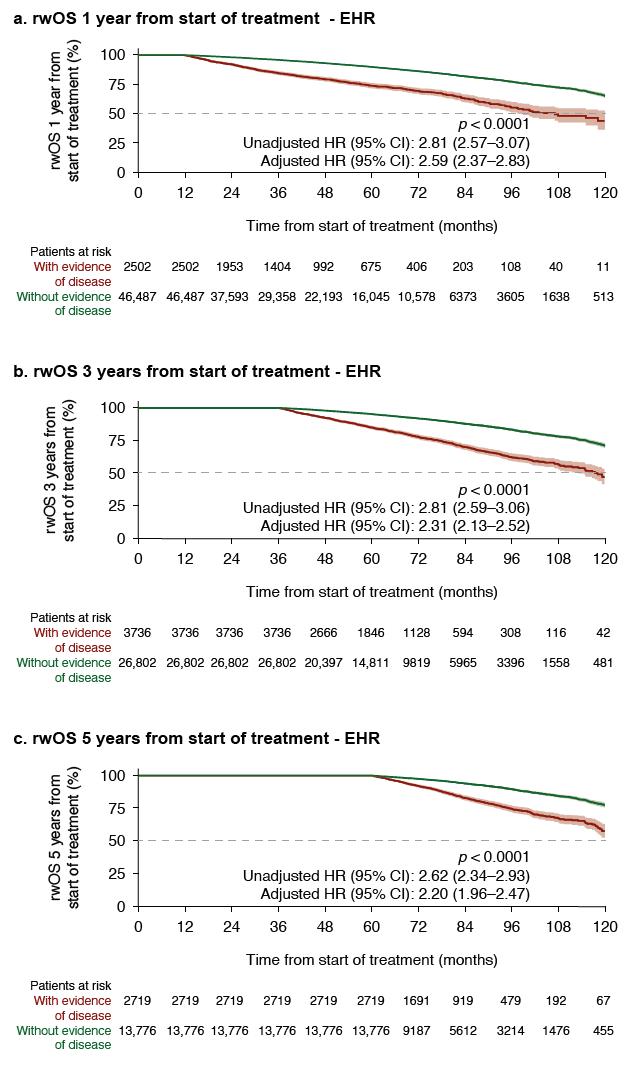


95% confidence intervals shown in shading around plotted lines

*CI* confidence interval, *EHR* electronic health records, *HR* hazard ratio, *LAPC* locally advanced prostate cancer, *LPC* localized prostate cancer, *rwOS* real-world overall survival
